# An Unusual Presentation of Esophageal Tuberculosis: A Case Report

**DOI:** 10.7759/cureus.29642

**Published:** 2022-09-27

**Authors:** Tafseer Zahra, Yuvapriya Ravikumar, Diana Voloshyna, Yumna Shams, Tanveer Ahamad Shaik, Qudsia I Sandhu, Saveeta Sahtiya, Faraz Saleem, Muhammad Abu Zar Ghaffari

**Affiliations:** 1 Medicine, California Institute of Behavioral Neurosciences & Psychology, Fairfield, USA; 2 Internal Medicine, Krishna Institute of Medical Sciences, Karad, IND; 3 School of Medicine, University of Michigan, Ann Arbor, USA; 4 Internal Medicine, Dow University of Health Sciences, Dow International Medical College, Karachi, PAK; 5 Cardiovascular Medicine, University of Louisville School of Medicine, Louisville, USA; 6 Medicine, Ghazi Khan Medical College Dera Ghazi Khan, Dera Ghazi Khan, PAK; 7 Internal Medicine, People's University of Medical & Health Sciences, Nawabshah, PAK; 8 Internal Medicine, Akhtar Saeed Medical & Dental College, Lahore, PAK

**Keywords:** git tb, endoscopy, git, esophageal tb, tb

## Abstract

The lungs, kidneys, liver, and pancreas are just some of the organs that can be affected by tuberculosis. Tuberculosis is a disease that can affect many organs of the human body. Rarely can tuberculosis (TB) manifest itself in the digestive tract; in fact, the gastrointestinal tract ranks as the sixth most common site of extrapulmonary TB. However, involvement of the esophagus by tuberculosis is extremely uncommon. We present a case of esophageal tuberculosis in a 27-year-old man with epigastric pain and weight loss as his only symptoms. There were no complaints of odynophagia or dysphagia, nor was there any evidence of immunodeficiency. Upper gastrointestinal endoscopy found an ulcer 26 centimeters from the incisor. Histopathology and a biopsy confirmed the diagnosis of primary esophageal tuberculosis. Six months after beginning anti-TB therapy, he was confirmed to be free of tuberculosis.

## Introduction

According to recent studies, tuberculosis (TB) is the largest cause of death worldwide from a single infectious disease agent [1]. In 2019, around 10 million people contracted tuberculosis worldwide, and an estimated 1.2 million HIV-negative people died from TB [2]. It affects the respiratory system predominately. Eleven percent of individuals with extrapulmonary TB had gastrointestinal (GI) involvement [2], with the terminal ileum, cecum, and peritoneum being the most common sites [3]. TB affecting the esophagus is one of the rarest forms of extrapulmonary TB, with a frequency of 0.2% [2]. Nonetheless, claims of esophageal tuberculosis (ET) have grown during the previous two decades. Generally, patients report dysphagia and/or odynophagia, and occasionally epigastric discomfort, chest pain, or hematemesis [1]. It can be caused by either primary involvement of the esophagus by tuberculosis or subsequent expansion from surrounding structures [3]. Due to many defensive mechanisms, such as the existence of mucus-coated stratified squamous epithelium, primary ET is extremely uncommon. Therefore, the majority of instances of ET are due to secondary infection from surrounding infected structures [4]. We discuss the example of a 27-year-old male who was diagnosed with an ulcer as a result of *E. coli*. This case report highlights the importance of suspecting tuberculosis in esophageal ulcers, especially in endemic regions such as Pakistan, in order to avoid complications such as perforation, bleeding, fistula formation, fatal hematemesis, traction diverticula aspiration pneumonia, and esophageal strictures.

## Case presentation

A 27-year-old South Asian male presented to the Outpatient Department of a tertiary care hospital with complaints of burning chest pain for the last three months. He had no history of difficulty swallowing or odynophagia. The pain usually begins after eating and has recently worsened, prompting him to seek medical attention. The patient reported low-grade intermittent fever for the last few weeks, with weight loss and loss of appetite. He has no history of smoking and has denied any alcohol intake. There was no cough or shortness of breath, and the patient denied any family history of chronic illness, including TB. The patient has worked as a construction worker for the last three years and has poor socioeconomic status. The patient took a self-medicated proton pump inhibitor (PPI) for the previous few weeks, but the pain seems to be worsening with time, with no relief.

On physical examination, the patient had a low-grade fever (101.1 degrees Celsius), stable vital signs, and a soft abdomen. His cervical lymphadenopathy was unremarkable. Extensive investigations were conducted, including a complete blood count (Cbc), erythrocyte sedimentation rate (ESR), sputum culture for acid-fast bacillus (AFB), hepatitis screening, and HIV screening. His Cbc revealed raised WBCs (13200) with lymphocytic predominance. His ESR was 105 mm in the first hour (Table 1).

**Table 1:** Laboratory investigations of the patient Hb: Hemoglobin; MCV: Mean corpuscular volume; ALT: alanine aminotransaminase; AST: aspartate aminotransferase; BUN: Blood urea nitrogen; Cr: Creatinine; ESR: Erythrocyte sedimentation rate; HbA1c: Hemoglobin A1C

Complete blood count parameters	Value
Hb (g/dL)	12.6 (13.5 - 17.5)
MCV (fl)	76.8 (80 - 100)
WBC (X10^9^/L)	13.2 (4.5 - 11)
Platelets (X10^3^/uL)	260 (150 - 400)
ALT (IU/L)	24 (7 to 55)
AST (IU/L)	33 (8 to 48)
BUN (mg/dL)	24 (6 to 24)
Cr (mg/dL)	1.1 (0.7 to 1.3)
ESR (mm/hr)	105 (1 to 13)
HbA1c%	5.5 (Below 5.7%)

**Figure 1:**
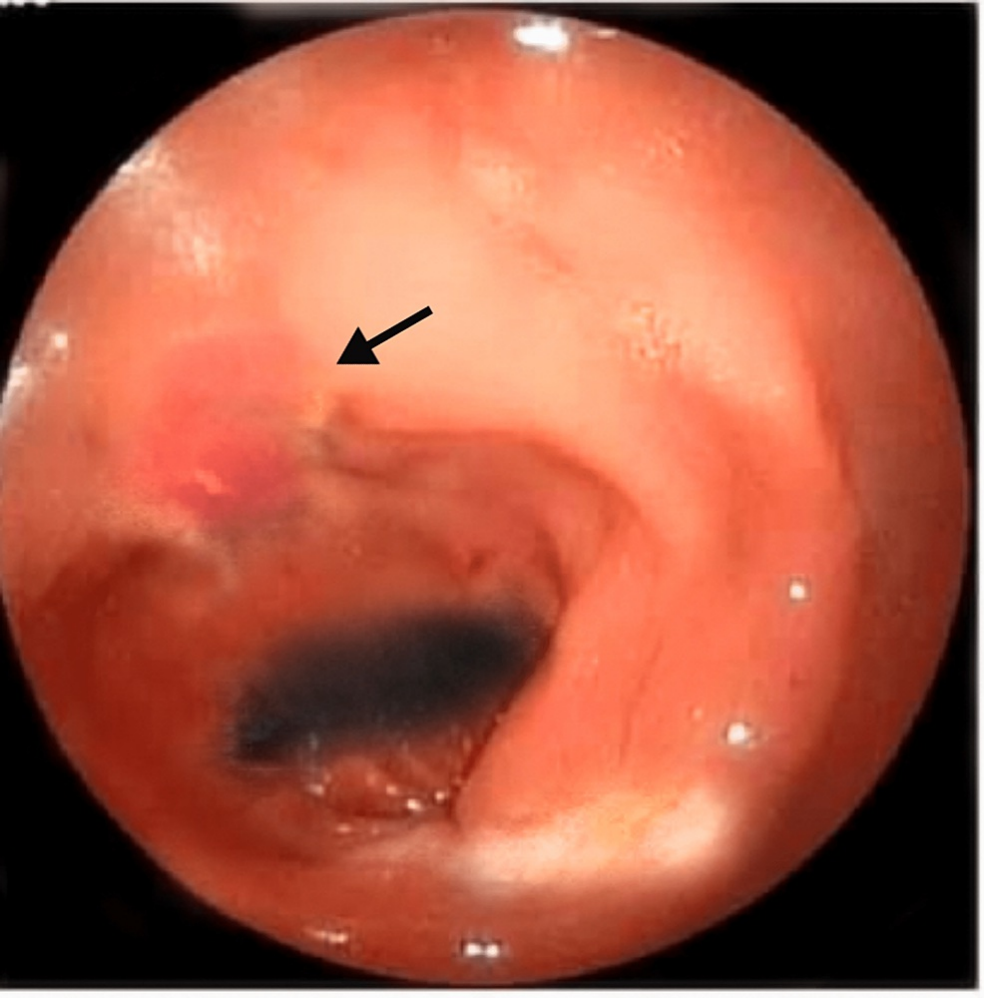
Endoscopy of esophagus showing ulcer

Gastroduodenoscopy was performed which revealed an ulcer (1.3x0.9 cm) in the distal one-third of the esophagus, with unremarkable findings in the stomach and duodenum Figure 1.

**Figure 2:**
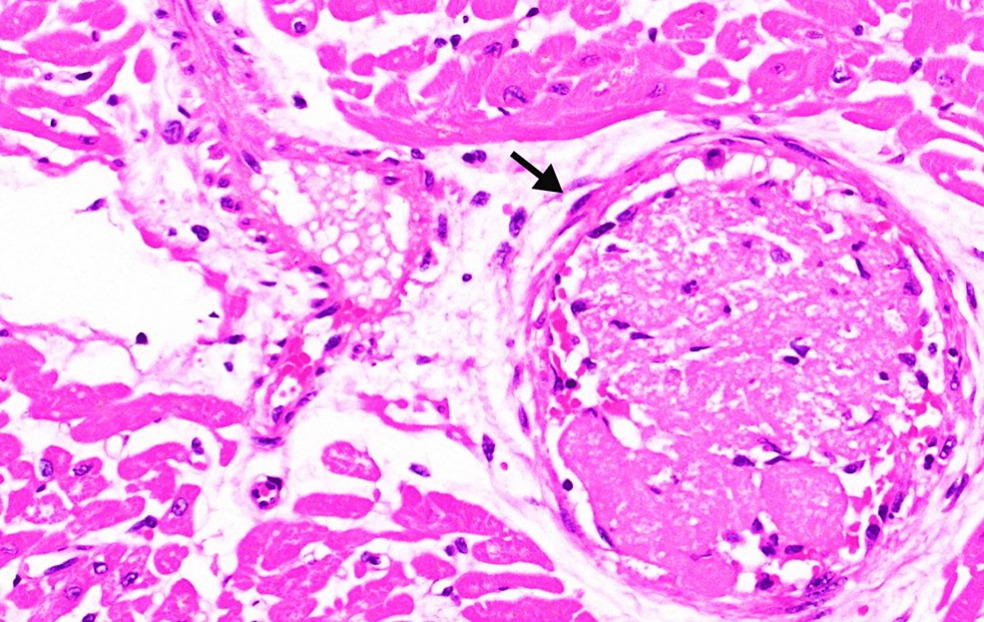
Caseating granuloma in the distal esophagus

The *H. pylori* gastric biopsy report was unremarkable. However, the biopsy of the lesion revealed a granulomatous caseating lesion with an eosinophilic background Figure 2.

**Figure 3:**
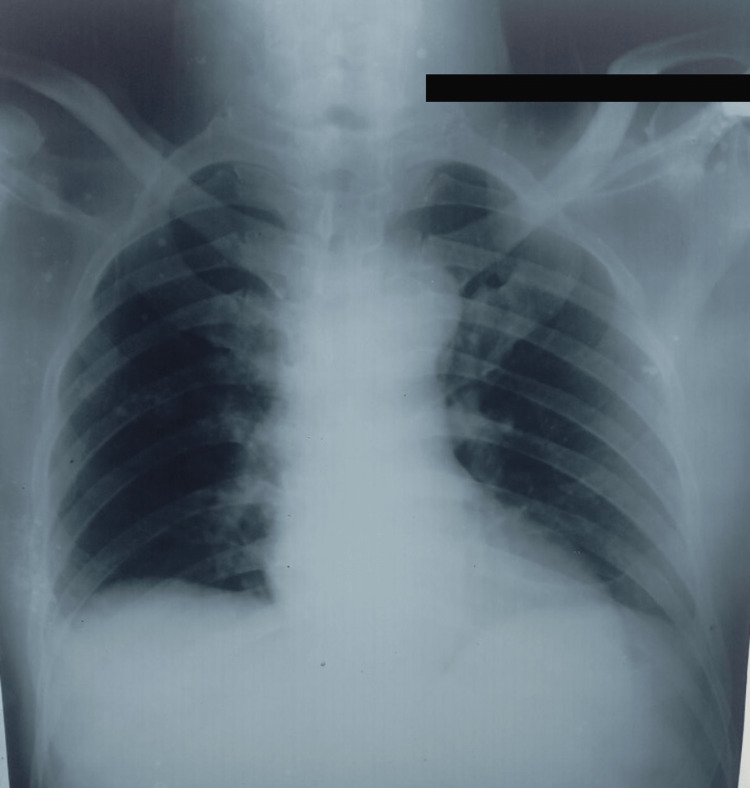
X-ray image of the patient

Upon suspicion of TB, a nucleic acid amplification test (NAAT) was performed, which was positive for tuberculosis. To rule out secondary causes of TB, a chest X-ray along with a CT chest and abdomen was carried out, however, the findings were unremarkable (Figure 3).

Hence, a provisional diagnosis of primary esophageal TB was made. The patient was counseled and was immediately started on an anti-tuberculous regimen. His treatment plan consisted of a four-month regimen of isoniazid, rifampin, pyrazinamide, and ethambutol, directly observed therapy (DOT). The patient was advised to follow up every three months. His family was also instructed to undergo screening for TB. Upon follow-up at six months the patient's upper GI endoscopy was unremarkable, indicating a complete recovery.

## Discussion

Extrapulmonary tuberculosis (EPTB) accounts for more than one-fifth of the worldwide disease burden. Among the numerous EPTB sites, GI TB is the sixth most prevalent, however very few cases occur in the esophagus [5]. TB of the esophagus often affects its middle section [6]. TB of the esophagus typically results from direct extension from surrounding structures, such as mediastinal lymph nodes and pulmonary sites [2]. It is hypothesized that the accumulation of swallowed sputum or infected milk products is one of the reasons for GI TB [7]. The patient's history of TB exposure and the disease's local incidence lead to a high index of suspicion in symptomatic patients. Contact with TB patients increases the likelihood of active TB infection. Typically, CT of the chest and abdomen is employed to evaluate the extent of gastrointestinal tuberculosis [8].

The severity of esophageal involvement dictates the severity of symptoms. Depending on inflammation of the mucosa, the development of ulcers or polypoid lesions, and constriction, the primary symptoms may differ. The esophageal lesion may also be complicated by chronic inflammation, diverticulum development, and infrequently tracheoesophageal fistula [9]. Aspiration pneumonia, esophageal strictures, esophagotracheal fistula, traction diverticula, esophagomediastinal fistula, fatal hematemesis, and amyloidosis are documented consequences of ET [10]. Dysphagia is the most prevalent esophageal condition, accounting for 90% of all esophageal TB cases, and it is always evaluated with an upper gastrointestinal tract endoscopy [11]. Dysphagia (84%) and odynophagia (42%), but rarely searing chest pain or epigastric pain (2%), were the most common presenting symptoms in a retrospective study of 24 patients with suspected ET [12].

It is uncommon for ET to present without dysphagia and/or odynophagia [13]. Our patient was HIV-negative and, with the exception of chest pain, exhibited no conventional TB symptoms. He had no radiological evidence of pulmonary TB. Neither the lungs nor the mediastinal lymph nodes were affected by tuberculosis. Since our patient did not exhibit any overt signs of pulmonary TB, we can reasonably conclude that he contracted ET through ingestion, making the diagnosis of primary ET. The time required for an infectious agent to adhere to the esophageal mucosa is likely to be significantly less than the time required for the pathogen to enter the body. However, it is unclear how this may occur, considering that the transit time is presumably insufficient for the human body [1]. Due to its rarity and similarity to other symptomatic esophageal illnesses, the clinical, radiological, and endoscopic characteristics of ET are not well defined [2,3]. Approximately 65% of patients with ET exhibit nonspecific chest radiographic abnormalities [3]. And the existence of granulomas with central caseous necrosis in a patient with a contact history is diagnostic.

Histopathology and tuberculosis-polymerase chain reaction (PCR) are the core tests for verifying the diagnosis of ET. Histology reveals an epithelioid granuloma with Langhans cells and caseous necrosis in the center. Classical granulomas are observed in only 50% of patients, while AFB is observed in 25%. According to Mokoena et al. endoscopic mucosal biopsies have a sensitivity of 22% [14]. The necrotizing granuloma biopsy findings and the incidence of tuberculosis in our region of the world lead us to the provisional diagnosis of TB in this patient. This was corroborated by a complete response to antituberculous treatment (ATT). TB was detected in a patient with caseating granulomata, which were proved to be *Mycobacterium tuberculosis* (MTB). The medication of choice for GI TB is standard anti-tubercular treatment; especially for tubercular strictures which improve substantially with anti-tubercular drugs alone. ET is initially treated for two months with isoniazid, rifampicin, pyrazinamide, and ethambutol, followed by 4 months of isoniazid, rifampicin, and ethambutol [15]. In GI TB, surgery is reserved for intestinal blockage, abscess, perforation, or fistula development that does not resolve [16].

This case study demonstrates the significance of adding ET in the differential diagnosis of epigastric and chest discomfort, especially in patients at risk for TB. To avoid missing a potential case of ET, it is advisable to include a differential that includes ET for patients who come with both types of pain.

## Conclusions

Esophageal tuberculosis can occur in the absence of clinical findings, historically associated with tuberculosis. There is a scarcity of information in the literature on the cases of isolated esophageal tuberculosis. Epigastric pain is rarely associated with esophageal tuberculosis and a refractory case should be tested by upper gastrointestinal endoscopy followed by a mandatory AFB test. ATT would lead to complete resolution and the prognosis is excellent. There is one point, however, to ponder upon since an association between tuberculosis and esophageal clinical findings has already been established in the literature, the known cases of extra-esophageal tuberculosis should be particularly tested for esophageal tuberculosis when present with the signs and symptoms of esophageal disease.
